# Reward maximization assessed using a sequential patch depletion task in a large sample of heterogeneous stock rats

**DOI:** 10.1038/s41598-023-34179-8

**Published:** 2023-04-29

**Authors:** Amy M. Gancarz, Suzanne H. Mitchell, Anthony M. George, Connor D. Martin, Marisa C. Turk, Heather M. Bool, Fahmida Aktar, Francis Kwarteng, Abraham A. Palmer, Paul J. Meyer, Jerry B. Richards, David M. Dietz, Keita Ishiwari

**Affiliations:** 1 Department of Psychology, California State University, Bakersfield, Bakersfield, CA 93311 USA; 2 Department of Behavioral Neuroscience, Oregon Health & Science University, Portland, OR 97239 USA; 3 Department of Psychiatry, Oregon Health & Science University, Portland, OR 97239 USA; 4 Oregon Institute for Occupational Health Sciences, Oregon Health & Science University, Portland, OR 97239 USA; 5 Clinical and Research Institute on Addictions, University at Buffalo, Buffalo, NY 14203 USA; 6 Department of Pharmacology and Toxicology, Jacobs School of Medicine and Biomedical Sciences, University at Buffalo, Buffalo, NY 14203 USA; 7 Department of Psychiatry, University of California San Diego, La Jolla, CA 92093 USA; 8 Institute for Genomic Medicine, University of California San Diego, La Jolla, CA 92093 USA; 9 Department of Psychology, University at Buffalo, Buffalo, NY 14260 USA

**Keywords:** Motivation, Reward

## Abstract

Choice behavior requires animals to evaluate both short- and long-term advantages and disadvantages of all potential alternatives. Impulsive choice is traditionally measured in laboratory tasks by utilizing delay discounting (DD), a paradigm that offers a choice between a smaller immediate reward, or a larger more delayed reward. This study tested a large sample of Heterogeneous Stock (HS) male (n = 896) and female (n = 898) rats, part of a larger genetic study, to investigate whether measures of reward maximization overlapped with traditional models of delay discounting via the patch depletion model using a Sequential Patch Depletion procedure. In this task, rats were offered a concurrent choice between two water “patches” and could elect to “stay” in the current patch or “leave” for an alternative patch. Staying in the current patch resulted in decreasing subsequent reward magnitudes, whereas the choice to leave a patch was followed by a delay and a resetting to the maximum reward magnitude. Based on the delay in a given session, different visit durations were necessary to obtain the maximum number of rewards. Visit duration may be analogous to an indifference point in traditional DD tasks. Males and females did not significantly differ on traditional measures of DD (e.g. delay gradient; AUC). When examining measures of patch utilization, females made fewer patch changes at all delays and spent more time in the patch before leaving for the alternative patch compared to males. Consistent with this, there was some evidence that females deviated from reward maximization more than males. However, when controlling for body weight, females had a higher normalized rate of reinforcement than males. Measures of reward maximization were only weakly associated with traditional DD measures and may represent distinctive underlying processes. Taken together, females performance differed from males with regard to reward maximization that were not observed utilizing traditional measures of DD, suggesting that the patch depletion model was more sensitive to modest sex differences when compared to traditional DD measures in a large sample of HS rats.

## Introduction

All animals face a simple, yet essential, choice between staying or leaving a resource in search of potentially better alternatives. Models of foraging behavior from behavioral ecology seek to understand choice behavior by assuming individuals maximize their net rate of energy gain when behaving optimally^1–3^. Patch leaving models specifically examine decisions about how much time a forager will devote to a dwindling resource (depleting patch) before departing to locate the next such resource. The Marginal Value Theorem (MVT)^3^ predicts that animals behaving optimally will leave when the rate of reward in the current patch equals the average rate of reward in the entire habitat. MVT predictions are based primarily on travel time between patches, which delays the availability of food in these alternative patches, but there are potentially other negative consequences associated with leaving the depleting patch that impact choice and postpone leaving. For example, animals risk predation, and the effort costs of traveling to an alternative patch may not vary linearly with travel time^4^. Foraging theorists have also suggested that food in alternative patches may be discounted because of the uncertainty about its availability and accessibility^5,6^. These considerations would suggest that foragers may stay longer than predicted by MVT in the depleting patch. Indeed, in many studies, animals exhibit this “overharvesting” behavior, operationally defined as a preference for smaller, more immediate rewards in a current patch relative to the potentially larger rewards available after a delay of traveling to a new patch. Conceptualized as such, overharvesting has been proposed to reflect “impulsive choice” in an intertemporal choice paradigm^7–14^.

In laboratory studies of behavioral economics, impulsive choice has also been studied using Delay Discounting (DD) tasks, in which individuals choose between smaller, sooner and larger, later rewards. A discounting function can be constructed to describe how choices change as a consequence of variations in the duration of the delay to the larger reward. The gradient of the function or area-under-the-curve function provides a metric of the extent to which delays affect choices. Performance on traditional DD tasks is both genetically^15^ and behaviorally associated with various aspects of the drug abuse continuum in humans^16–23^, as well as numerous other psychopathologies including attention deficit hyperactivity disorder^24^. Indeed, several researchers have suggested that the excessive devaluation of delayed rewards, as assessed by DD tasks, is a transdiagnostic feature of psychiatric disorders^25^.

Choice behavior in both patch foraging and DD tasks may tap similar reward valuation processes that drive individuals to maximize reward while also devaluating rewards that are not currently present (delayed rewards in alternative food patches). However, few studies have simultaneously measured both reward maximization and DD processes in a single task. Accordingly, we examined choice behavior in a large sample of males and females, using the sequential patch depletion procedure^26^, to evaluate the extent that behavior on the sequential patch depletion task maximized the net rate of reinforcer gain, as derived from the MVT. In addition, we also investigated the degree to which behavior could be described using traditional DD metrics (gradient of the DD function, area under the discounting curve). Male and female outbred Heterogeneous Stock (HS) rats, known for their high genotypic and phenotypic variability^27,28^, chose between a smaller, sooner reward by staying in a rapidly depleting patch and a delayed but larger reward by investing the time needed to change to a non-depleted patch. The large sample size provides the power to identify subtle effects and to the best of our knowledge, will provide an in-depth analysis of behavior in this the first investigation of performance in HS rats in a study of patch leaving.

## Results

### Delay discounting

**Figure 1:**
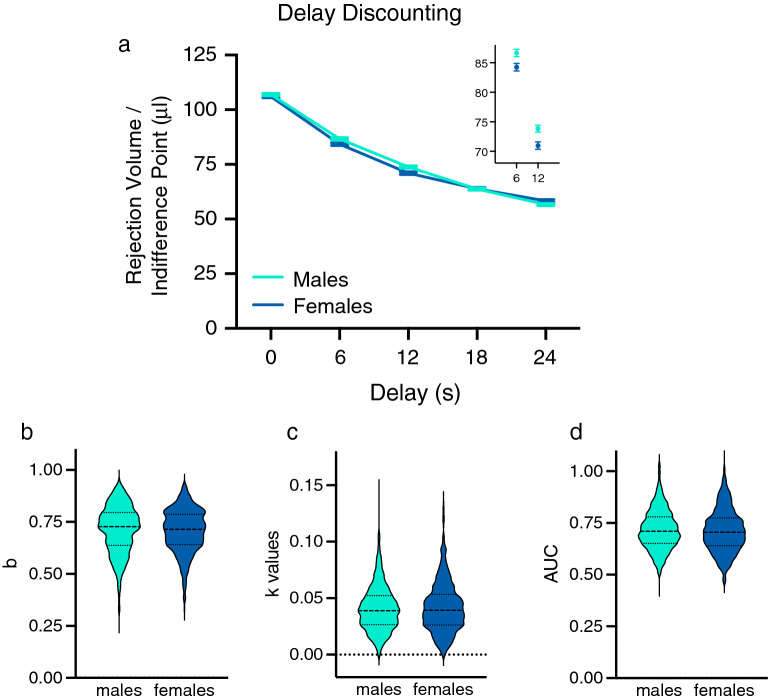
Delay discounting (**a**) This plot shows the mean (± SEM) indifference point (rejection volume of water) as a function of delay. Inset graph highlights the differences between sexes at delays of 6 and 12 s. Data are the mean (± SEM) of the last two test sessions per delay. (**b**–**d**) The outlines of the violin plots illustrate the average kernel probability density (i.e., the width of the colored area represents the proportion of the data for that variable). The dashed lines indicate the median data, and dotted lines indicate quartiles. (**b**) This plot represents the bias parameter, *b* for one response alternative. No significant difference between sexes was observed. (**c**) This plot represents the discount parameter, k, a free parameter that indicates the rate of reinforcer devaluation as a result of the delayed delivery of the reward. The k values of the best fitting hyperbolic discount functions indicated that sex did not affect delay discounting (DD). (**d**) This plot represents the normalized area under the curve (AUC) of the indifference point as a function of delay (ratio). No significant difference between sexes was observed. *p < 0.05, **p < 0.01, ***p < 0.001 between males (n = 896) and females (n = 898).

Performance in the Sequential Patch Procedure was first analyzed using a traditional DD approach, which focused on the rejection volumes at the different change over delay (COD) travel times (Fig. 1a). Sex differences were insufficiently large to be reflected in the hyperbolic discounting function gradient (*k;* Fig. 1c) or AUC (Fig. 1d). The lack of difference at 0 s indicates that males and females do not differ on the bias parameter of the hyperbolic equation (*b*; Fig. 1b).

### Patch utilization

**Figure 2:**
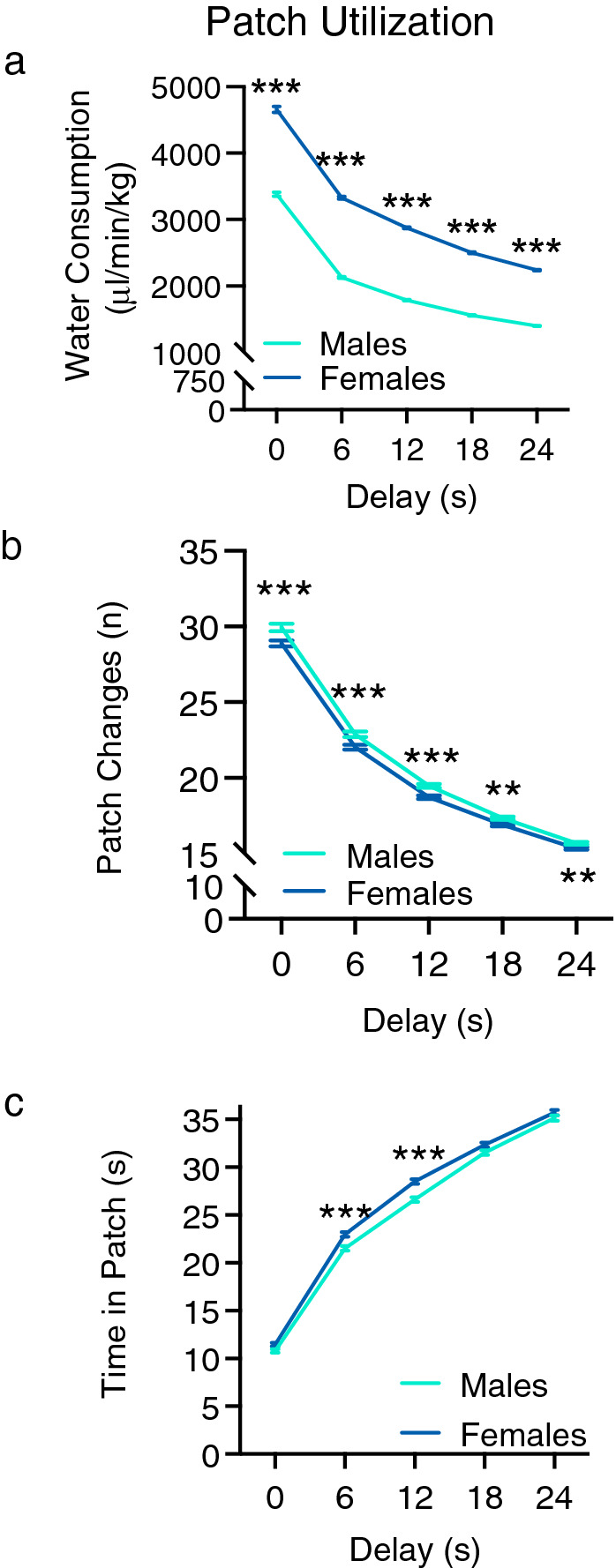
Patch utilization. These data show the performance of heterogeneous stock rats on the sequential patch depletion procedure across various delays (0, 6, 12, 18, & 24 s). Aqua lines represent male HS rats and blue lines represent female HS rats. (**a**) Water consumption (μl/min/kg) across all delays. Females had significantly higher water consumption relative to males when body weight was controlled for at all delays tested. (**b**) Number of patch changes in the last two sessions. Females switched patches significantly fewer times (made fewer “leave” choices) than males for all delays. (**c**) Mean time spent in the patch (snout poke receptacle) before leaving for the alternate patch. Females stayed in the patch significantly longer at 6- and 12-s delays. Data are expressed as the average ± standard error; *p < 0.05, **p < 0.01, ***p < 0.001 between males (n = 896) and females (n = 898).

Males and females displayed widespread differences in resource acquisition. Water consumption was evaluated by controlling for body weight^29–31^. A two-way between-subject ANOVA with sex as the between-subject factor and delay (0, 6, 12, 18, and 24 s) as the within-subject factor was conducted to determine if sex moderated water consumption (µL/min/kg). There was a significant interaction between delay and sex [F(4,7168) = 60.502, p < 0.000, **η**_**p**_^**2**^ = 0.033] and significant main effects of delay [F(4,7168) = 5672.762, p < 0.000,** η**_**p**_^**2**^ = 0.760] and sex [F(1,1792) = 2971.919, p < 0.000, **η**_**p**_^**2**^ = 0.624]. Independent samples *t* test with Bonferroni correction (p = 0.01) identified that controlling for body weight, females had a significantly greater rate of water reinforcement at delays of 0 – 24 s [D0: t(1792) = − 24.278, p < 0.001, *d* = − 1.146; D6: t(1792) =  − 49.142, p < 0.001, *d* = − 2.320; D12: t(1792) =  − 58.717, p < 0.001, *d* = − 2.773; D18: t(1792) =  − 65.481, p = 0.001* d* = − 3.092; D24: t(1792) =  − 66.447, p = 0.001 *d* = − 3.136] compared to males (Fig. 2a). This sex difference was consistent when the normalized AUC was determined for the water consumption data with females having significantly greater AUC compared to males [t(1792) =  − 59.779, p < 0.001, *d* = − 2.823; Supplementary Fig. 1a].

These effects were driven by differences in several associated variables. A two-way between-subject ANOVA examining the number of patch changes/leave choices identified a significant delay x sex interaction [F(4,7168) = 3.805, p < 0.01] and significant main effects of delay [F(4,7168) = 5387.924, p < 0.001] and sex [F(1,1792) = 15.933, p < 0.001]. Effect sizes (**η**_**p**_^**2**^) were as follows: delay = 0.75; sex = 0.09. Post-hoc pairwise comparison Bonferroni tests showed that patch changes were significantly different for each delay, indicating that the number of times the rats changed patches decreased as the delay increased. Independent samples t tests with Bonferroni corrections showed that females exhibited significantly fewer patch changes (leave choices) than males at all delays tested [0 s, t(1792) = 3.293, p < 0.001, *d* = 0.156; 6 s, t(1792) = 3.366, p < 0.001, *d* = 0.159; 12 s, t(1792) = 4.082, p < 0.001, *d* = 0.193; 18 s, t(1792) = 2.869, p = 0.004, *d* = 0.135; 24 s, t(1792) = 2.840, p = 0.005, *d* = 0.134] (Fig. 2b). This sex difference was consistent when the normalized AUC was determined for patch changes data with males having significantly greater AUC compared to females [t(1792) = 4.007, p < 0.001, *d* = 0.189; Supplementary Fig. 1b]. The difference in number of patch changes was accompanied by sex differences in the mean amount of time spent in patches; a two-way between-subject ANOVA revealed a significant interaction between delay and sex [F(4,7168) = 5.710, p < 0.001] and significant main effects of delay [F(4,7168) = 6456.707, p < 0.001] and sex [F(1,1792) = 15.285, p < 0.001]. Effect sizes (**η**_**p**_^**2**^) were as follows: delay = 0.783; delay x sex = 0.000; sex = 0.008. Post-hoc pairwise comparison Bonferroni tests showed that the amount of time in patch was significantly different for each delay, indicating that rats increased the amount of time spent in the patch as the delay increased. Independent samples t tests with Bonferroni corrections revealed that females spent significantly more time in the patch than males before leaving for a new patch at 6- and 12-s delays [6 s, t(1792) =  − 4.312, p < 0.001, *d* = − 0.204; 12 s, t(1792) =  − 5.375, p < 0.001, *d* = − 0.254] (Fig. 2c). This sex difference was consistent when the normalized AUC was fit to the data with females having significantly greater AUC compared to males [t(1792) =  − 4.221, p < 0.001, *d* = − 0.199; Supplementary Fig. 1c]. Taken together, these data indicate sexually dimorphic performance on the characteristics of patch visits on the sequential patch depletion task, with females making fewer patch changes, remaining in a patch for longer periods of time resulting in higher normalized rate of reinforcement.

**Figure 3:**
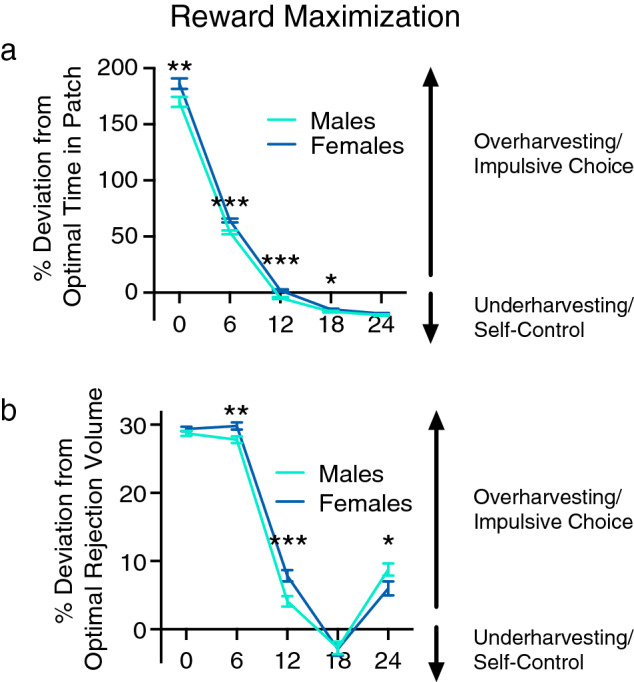
Reward maximization (**a**) Percent deviation from optimal time in patch. Observed time in patch was compared to the optimal stay time for reward maximization. Positive values indicate staying longer than optimal, whereas negative values indicate leaving before optimal. Male heterogenous stock rats showed significantly greater reward optimization at delays of 0–6 s, whereas female rats showed significantly greater reward maximization at delays of 12–18 s. (**b**) Percent deviation from optimal rejection volume. Observed rejection volume (indifference point) was compared to the optimal rejection volume for reward maximization. Positive values indicate a rejection volume less than optimal, and negative values indicate a rejection volume greater than optimal. Males showed significantly greater reward optimization at delays of 6 and 12 s, whereas females showed significantly greater reward maximization at delay of 24 s. Data are expressed as the average ± standard error; *p < 0.05, **p < 0.01, ***p < 0.001 between males (n = 896) and females (n = 898).

To assess whether these patch utilization patterns generally reflected a reward maximization strategy (according to the MVT), we assessed differences in deviations from the optimal time in patch; a two-way between-subject ANOVA identified a significant interaction between delay and sex [F(4,7168) = 4.83, p < 0.001] and significant main effects of delay [F(4,7168) = 3478.69, p < 0.001] and sex [F(1,7192) = 13.293, p < 0.001]. Effect sizes (**η**_**p**_^**2**^) were as follows: delay = 0.660; delay x sex = 0.003; sex = 0.007. As can be seen in Fig. 3a, rats spent less time in the patch than predicted by our reward maximization calculation when the CODs were short, but this deviation declined systematically until the time in patch approximated optimal predictions (COD of 12 s) and then exceeded predictions toward underharvesting (leaving the patch sooner than optimal). Post-hoc independent samples t tests with Bonferroni corrections revealed that the deviation from the optimal time in patch was more extreme for females than males at delays of 0–6 s [0 s, t(1792) =  − 2.485, p < 0.01,* d* = − 0.117; 6 s, t(1792) = 4.312, p < 0.001, *d* = − 0.204], whereas males deviated from optimal more at longer delays [12 s, t(1792) =  − 5.375, p < 0.001, *d* = − 0.254; 18 s, t(1792) =  − 2.74, p = 0.015, *d* = − 0.103] (Fig. 3a).

**Figure 4:**
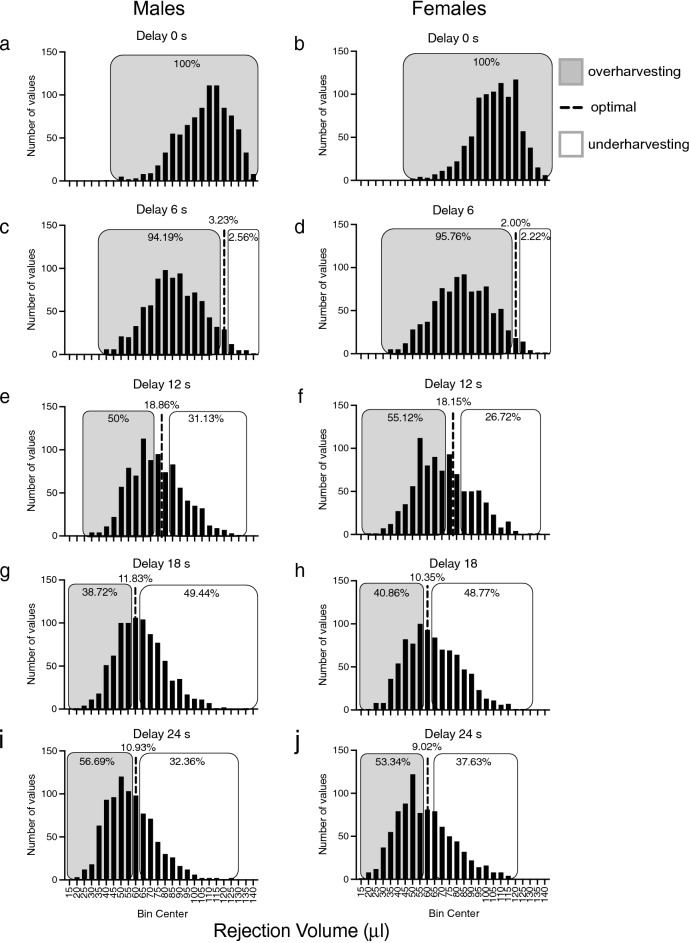
Frequency distribution of rejection volumes for male and female rats across all delays tested. The dotted lines indicate the percentages of animals exhibiting optimal reward maximization. Gray shaded regions represent the percentages of animals exhibiting overharvesting, whereas the open boxes represent the percentages of animals exhibiting underharvesting at delays of 0 s ((**a**) males; (**b**) females), 6 s ((**c**) males; (**d**) females), 12 s ((**e**) males; (**f**) females), 18 s ((**g**) males; (**h**), females), and 24 s ((**i**), males; (**j**), females). n = 896 (males) and 898 (females).

To further evaluate reward maximization, the observed rejection volume was compared to the optimal rejection volume (predicted by the MVT); a two-way between-subject ANOVA identified a significant interaction between delay and sex [F(4,7168) = 10.109, p < 0.001] and a significant a main effect of delay [F(4,7168) = 1428.742, p < 0.001]. Effect sizes (**η**_**p**_^**2**^) were as follows: delay = 0.444; delay x sex = 0.006. Females deviated from the optimal rejection volume significantly more than males at delays of 6 and 12 s [6 s, t(1792) =  − 2.672, p < 0.001, *d* = − 0.126; 12 s, t(1792) =  − 3.287, p = 0.005,* d* = − 0.155], whereas males deviated significantly more than females at 24 s [t(1792) = 2.032, p < 0.01,* d* = 0.096] (Fig. 3b). Relative to MVT predictions, both sexes were exhibiting overharvesting (leaving the patch after it would be considered optimal) at 0- and 6-s delays, but at longer delays (12–24 s), there was a shift toward optimal. This altered patch leaving as a function of COD is clearly illustrated by changes in the frequency distributions of rejection volumes across delays; the percentages of animals overharvesting decreased as delay increased (Fig. 4). These data indicate that rats’ behavioral choices deviate from the values predicted by the MVT.

### Strengths of associations between patch utilization, reward maximization, and DD

Table 1 shows the correlations amongst traditional DD composite variables (*k* and AUC), patch utilization composite variables (patch changes, time in patch, and rate of water reinforcement), and reward maximization composite variables (time and volume deviations) in male and female rats. For both sexes, there were strong significant negative associations between k and AUC values (*p* values < 0.001; Table 1). DD composite variables significantly correlated with the composite scores for patch changes for males and females (p values < 0.05). However, while significant associations were observed between patch utilization composite variables (time in patch, patch changes) and DD composite variables (*k* and AUC; all p values < 0.05), these associations appeared weaker than the associations observed between patch utilization variables and reward maximization variables (time and volume deviation; all p values < 0.05). Weak to no associations were observed for both DD and reward maximization and rate of water reinforcement (r = 0.3 < 0.03, see Table 1).

**Table 1:** Pearson correlation matrices for male and female rats.

	AUC	VD	TD	TIP	PC	WR
Males (*n* = 896)						
*k*	− 0.76**	0.56**	− 0.05	0.32**	− 0.30**	0.32**
AUC		− 0.56**	− 0.27**	− 0.50**	0.45**	0.05
VD			− 0.79**	− 0.86**	− 0.90**	0.12**
TD				− 0.92**	0.87**	0.20**
TIP					− 0.89**	− 0.19**
PC						0.08*
Females (*n* = 898)						
*k*	− 0.73**	0.64**	− 0.13**	0.43**	− 0.35**	0.18**
AUC		− 0.59**	0.29**	− 0.52**	0.47**	0.18**
VD			− 0.75**	0.86**	− 0.88**	0.07*
TD				− 0.89**	0.88**	0.21**
TIP					− 0.90**	− 0.17**
PC						0.15**

*k* free parameter discounting index, *VD* volume deviation, *TIP* time in patch, *PC* number of patch changes, *WR* water reinforcement (μl/min/kg).

*Correlation is significant at the 0.05 level.

**is significant at the 0.01 level (two-tailed analysis).

To compare the strength of the associations between DD variables and reward maximization for each of the patch utilization variables, we applied the method described by Meng et al.^32^, which compares sets of non-independent overlapping correlation coefficients via a z-test procedure. For most comparisons, there were differences in the strength of associations (see Table 2 for z-scores). Correlations between patch utilization composite scores and reward maximization were significantly stronger than correlations between patch utilization and DD variables in both males and females (Tables 1 and 2). For example, correlation coefficients between number of patch changes and k (males: r = – 0.30; females: r = – 0.35) were significantly weaker than correlation coefficients observed between number of patch changes and time deviation (males: r = 0.87; females: r = 0.88; see Table 2 for z-scores). Similarly, correlation coefficients between number of patch changes and AUC (males: r = 0.45; females: r = 0.47) were significantly weaker than correlation coefficients observed between number of patch changes and time deviation (males: r = 0.87; females: r = 0.88; see Table 2 for z-scores). This pattern in the strength of associations was repeated for time in patch (Patch utilization correlation coefficients: Reward maximization > DD, as shown in Table 1). A different pattern emerged for normalized rate of water reinforcement. Here, both DD and reward maximization variables were either weakly or not significantly correlated to normalized rate of water reinforcement. Time deviation correlation coefficients were similar to those observed for k, and AUC correlation coefficients were similar to correlation coefficients for volume deviation. These results were confirmed using correlation comparison approaches in the R package^33,34^. Taken together, these data indicate that while standard DD variables are associated with patch utilization, these associations are weak relative to associations between patch utilization and reward maximization in both males and females.

**Table 2:** z-scores for relative strengths of correlations between delay discounting and reward maximization metrics in male and female rats.

Comparison	z-score		
	TIP	PC	WR
Males			
*k* vs. VD	42.97** (VD)	30.33** (VD)	6.56** (k)
*k* vs. TD	39.58** (TD)	− 32.81** (TD)	2.61** (k)
AUC vs. VD	12.58** (VD)	33.10** (VD)	− 1.19
AUC vs. TD	19.49** (TD)	− 15.90** (TD)	− 2.85** (TD)
Females			
*k* vs. VD	− 23.43** (VD)	28.63** (VD)	3.91** (k)
*k* vs. TD	37.44** (TD)	− 34.65** (TD)	− 0.61
AUC vs. VD	− 31.36** (VD)	31.63** (VD)	1.87
AUC vs. TD	18.80** (TD)	− 19.13** (TD)	− 0.77

*TIP* time in patch, *PC* number of patch changes, *WR* water reinforcement (μl/min/kg), *k* free parameter impulsivity index, *VD* volume deviation, *TD* time deviation, *AUC* area under the curve for indifference points.

Significant difference between correlation coefficients using the z-test procedure (*p < 0.05; **p < 0.01) outlined by Meng et al. (1992) and the R Cocur package (Diedenhofen & Musch, 2015). Variable in parentheses indicates statistically stronger correlation in comparison.

## Discussion

The Marginal Value Theorem (MVT) suggests that optimal foragers will choose to stay in a patch until the rate of reward falls below the average rate of reward in alternative patches^3^. Consequently, longer travel delays between patches make staying in a depleting patch a more optimal choice because the longer delay depresses the average rate of reward, resulting in foragers remaining in the patch for a longer period of time. Alternatively, shorter travel delays between patches makes staying in a depleting patch for a long period of time a less optimal choice, resulting in “leave” choices occurring sooner. This is supported by the current experiment, in which CODs (representing travel delays) altered choice behavior. Increasing delays resulted in a decrease in the number of patch changes and an increase in time in patch as rats made more “stay” choices.

The sequential patch depletion task is a valuable paradigm to understand important processes underlying optimal behavior because it has strong ethological validity. However, another important strength of this paradigm is the ability to examine reward maximization in greater depth. By assessing the percent deviation from optimality, which normalizes the data to map reward maximization for various delays, we found that animals tended to overharvest at short delays, as indicated by remaining in a patch longer than optimal. Conversely, rats underharvested when the delays were longer, as indicated by animals leaving the patch sooner than optimal. Notably, the greatest deviation from the optimal time in patch occurred when delays were < 6 s, that is 0 s. These data may imply that despite the absence of a travel cost, some other unidentified variable influences the rat’s choice to stay in the current patch. While the focus of MVT is about travel costs, other factors can also contribute to choices to stay in a patch, such as predation and energy expenditure^1,2,4^. In the current study, effort needed to change patches may have been weighted more at shorter delays. Overharvesting observed at shorter delays may be due to the nature of the sequential patch procedure. In this task, the reward is presented and there is a 4 s delay prior to presentation of the subsequent reward. Part of this time is spent consuming the initial reward, so it may be the case that the rewards are presented close enough together in time that the delay is not noticed until the volume is substantially lessened from the original volume. It is unclear if these variables are contributing to the sex differences found in this study. Future research is needed to tease apart how these factors may contribute to overharvesting.

Our data demonstrate that foraging behavior and traditional measures of impulsive choice, such as those provided by traditional DD indices, may share some characteristics of behavioral responding but may more strongly reflect distinct behavioral processes. We observed significant associations between DD and reward maximization variables. However, of note, reward maximization variables were more strongly associated with patch utilization variables than the DD variables. Consistent with this, Hayden, Pearson, & Platt^9^ showed that the performance of monkeys in a foraging task fit better with the MVT model than with hyperbolic DD function; they obtained a similar result when testing a patch-leaving foraging task interleaved with a traditional DD task^35^.

### Sex differences in DD, patch utilization and reward maximization

Greater DD, and by extension impulsive choice, is indicated by longer stays in a depleting patch with smaller rewards. Although females had lower indifference points than males at some delays, no significant differences in hyperbolic k values or AUC were found. Relatively few animal studies have examined sex differences in DD, however these studies have produced conflicting results. In animals, larger DD has been found in female rats^36,37^ and mice^38^, whereas others have identified larger DD in male rats^39,40^, and either age-dependent sex differences^41^, or no sex differences^42–45^ have been described. Similar discrepancies have been reported in some human studies, with greater discounting observed in women^15,23,46,47^, whereas other studies have found greater discounting in men^48–50^. Even more studies of humans have found no differences^51–58^. These disparate findings call for a more comprehensive examination of these differences in impulsive choice, as a foundation on which to explore the interrelationships between gender and DD in humans as well as their roles in psychopathologies (e.g. substance abuse).

In the present study, while both sexes deviated from optimal performance, females were less successful in reward maximization at relatively short delay times (i.e., decision-making about time spent in patch and the amount of reward to reject), though when controlling for body weight, an inverse relationship is observed with females exhibiting a higher rate of reinforcement despite having made fewer patch changes and spending more time in patches than males. The effect was only observed as a function of body weight and this pattern suggests that the relationship between normalized reinforcement rate and reward maximization is complex. The current sequential patch depletion task was not designed in a way that the normalized reinforcement rate would be greater if the time spent in patch and the amount of reward rejected were closer to the optimizing strategy. Future studies should design a suitable task and adjust the procedure to further dissect the sex differences observed in the present study to reconcile these inconsistencies.

These data are related to findings of sex differences in humans in the Iowa Gambling Task, in which men had a greater preference for cards that were advantageous in the long-term compared to women^59^. From this, van den Bos^59^ posited that males tend to focus more on long-term goals, shifting from exploration to exploitation, whereas females exhibited greater exploratory behavior^38,60^. The findings from the current study offer nuances to this hypothesis. When controlling for body weight, the female rats in our study had a greater rate of reinforcement than males, which does not support the interpretation that the males focused on long-term goals. Furthermore, females did not exhibit greater exploratory behavior as they made fewer patch changes and stayed in the patch significantly longer than males. Instead, the greater rate of switching between patches observed in males may reflect greater behavioral flexibility, whereas females may be demonstrating more perseverative behavior^61^. Reports of sex differences in perseverative behaviors have been inconsistent^62–65^, and thus, further research is needed to reconcile these discrepancies. This is of particular importance because perseveration is a feature of psychological disorders (e.g. schizophrenia, autism, OCD and drug addiction)^66^, and understanding these processes may have implications in sex-dependent vulnerability.

There are a variety of possible explanations for why females stayed longer in a patch. For example, there are sex differences in energy expenditure^67,68^, that may result in a female bias to preserve energy. Greater “overharvesting” observed in female rats at short delays may be an inaccurate interpretation: females may have exhibited appropriate levels of harvesting given environmental or biological factors, such as those related to reproductive success which made staying in the patch a more advantageous choice. Furthermore, their strategy may be to fully deplete the patch and exploit all resources before moving on to the next patch, as overharvesting may not abrogate visiting the patch in the future when resources have been replenished. In addition, females may have a differential sensitivity to tracking a changing environment, or to cues of change^69^. Indeed, Tropp & Markus^70^ found males and females utilize cues differently in various environments. When animals are presented with diminishing rewards upon the choice to stay in a patch, not only are they being reinforced, but they are also gaining new information about the quality of the reward, an important component of the economics of choice behavior^1,2^. Finally, females may tend to choose safer options, which is supported by studies in rats^71–74^.

Taken together, these data suggest that patch depletion model and by extension reward maximization was more sensitive to modest sex differences, compared to traditional DD tasks and metrics.

### Future directions

While we report differences between males and females in reward maximization, it is important to note we did not track estrous cycles in the females, so we cannot determine if this affected their performance on this task. To date, there is little evidence regarding the hormonal role in mediating these behavioral processes. Here, we present statistically different, but admittedly modest sex differences in performance on the sequential patch depletion task. This may be, in part, due to the role of the estrous cycle in performance on these tasks. In keeping with the literature exploring sex differences on DD, there is limited and conflicting evidence for the role of the estrous cycle on DD^36,75,76^. Future research is needed to determine the impact of the estrous cycle on performance in the patch depletion procedure.

The sequential patch model assumes that foragers have perfect knowledge of the model’s parameters, identified by Stephens^2^ as the “complete information assumption.” While the rats in our study had substantial training, this assumption may influence how we interpret their behavior. The differences between rewards associated with optimal rejection volumes were small, which may have contributed to the deviations from optimality. This issue has been noted previously in analyses of optimal performance on progressive schedules with reset^77–79^.

This is the first report of sex differences in a large sample of Heterogenous Stock rats tested using the sequential patch depletion procedure. By using a large sample of Heterogenous Stock rats, we showed that females performance differed from males with regard to reward maximization that were not revealed utilizing traditional measures in DD. Notably, frequency distributions of rejection volume indicate that there was also sizeable variability in rejection volumes within each sex, which enables us to explore individual differences or genetic/environmental variables that contribute to performance in this task in future studies. Furthermore, measures of reward maximization were only weakly associated with DD variables and thus may be mediated by different underlying processes. Taken together, these data show the utility in the use of the sequential patch depletion procedure for measuring choice and may have implications in vulnerability in the development of psychiatric diseases.

## Materials and methods

### Subjects

Male (n = 896) and female (n = 898) heterogeneous stock rats (HS; NMcwiWfsm:HS; RRID #RRID:RGD_13673907)^80^ tested in this study were part of the NIDA Center for GWAS in Outbred Rats (Principal Investigator, Dr. Abraham Palmer) and obtained from colonies maintained by Dr. Leah Solberg Woods [2015–2018: Medical College of Wisconsin: males n = 815, females n = 816; 2019: Wake Forest University: males n = 81; females: n = 82]. Experiments were conducted in batches of approximately 100 rats (4- to 5-months of age) at 3- to 4-month intervals. Rats were quarantined for 1- to 2-weeks upon arrival to University at Buffalo before being transferred to colony housing. The colony room was maintained at a constant temperature (22 ± 1 °C), humidity range (~ 55% ± 5%), and lights were on a reverse cycle (lights on from 19:00 to 07:00).

Rats were housed in same-sex pairs in plastic cages (42 × 22 × 19 cm) lined with bedding (Aspen Shavings). Prior to the start of the experiment, rats (n = 1590) were first tested on four behavioral tasks (social reinforcement, locomotor response to novelty, light reinforcement, and choice reaction time; data are not reported here). A subset of animals (n = 204) were not tested on the social reinforcement test because it was introduced after the first two batches had already been tested. At the onset of data collection, the mean (± SEM) age of the rats was postnatal day 136.58 ± 0.29 and weight was monitored over the course of the experiment.

Behavioral testing was conducted 6 days/week (Monday through Saturday) during the dark phase of the light–dark cycle between the hours of 08:30 and 12:30. Food (Teklad Laboratory Diet #8604) was available ad libitum in the home cages. Access to water was restricted to 30 min immediately following testing on Monday through Friday. At the end of the testing on Saturday, animals were given free access to water until approximately 12:30 h on Sunday (approximately 20 h prior to testing on Monday).

This study was conducted in accordance with protocols approved by the Institutional Animal Care and Use Committee at the University at Buffalo, and animals were treated in compliance with the Guide for the Care and Use of Laboratory Animals, and the study is reported in accordance with the ARRIVE guidelines^81^.

### Apparatus

**Figure 5:**
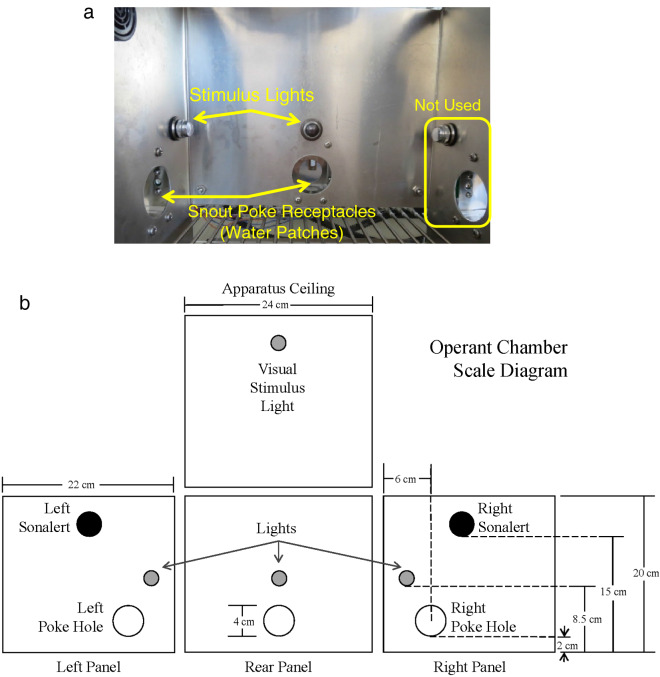
Illustration of the operant chamber used to measure performance in the sequential patch depletion procedure. (**a**) Photograph of the apparatus used in these studies. (**b**) Scaled diagram of the chamber. Images edited/modified using Microsoft Powerpoint.

Testing occurred in 24 locally constructed operant chambers (24 × 22 × 20 cm; Fig. 5) housed in sound-attenuating cabinets (Model # 3,000,000,187, Coleman, Wichita, KS), which were previously described in detail^82^. Briefly, the chambers had stainless-steel rod floors, aluminum back and side walls, and a Plexiglas front wall and top. Each test chamber had three snout poke receptacles (4 cm in diameter) located in the back and side walls. Infrared photobeam detectors, located 1 cm from the snout poke receptacle entrance, were used to record snout pokes. Three stimulus lights were located above each snout poke receptacle, and a fourth light was located in the ceiling of the test chamber. A Sonalert tone generator (SC628EJR; Mallory Sonalert Products, Indianapolis, IN) mounted on the right wall provided a pulsed 1.9-kHz tone. Acrylic dishes were located inside the left and rear snout poke receptacles and were connected to Tygon tubing, which delivered precise amounts of water from 60 ml syringes mounted on two single-speed syringe pumps (3.33 rpm, PHM-100; Med Associates, St. Albans, VT) external to the sound-attenuating cabinet.

The apparatus was controlled by MED-PC IV software (RRID:SCR_012156) with 1 ms temporal resolution running on computers with Microsoft Windows operating systems. Equipment was tested before test sessions and following any session in which a rat earned fewer than 30 reinforcers.

### Sequential patch depletion procedure

**Figure 6:**
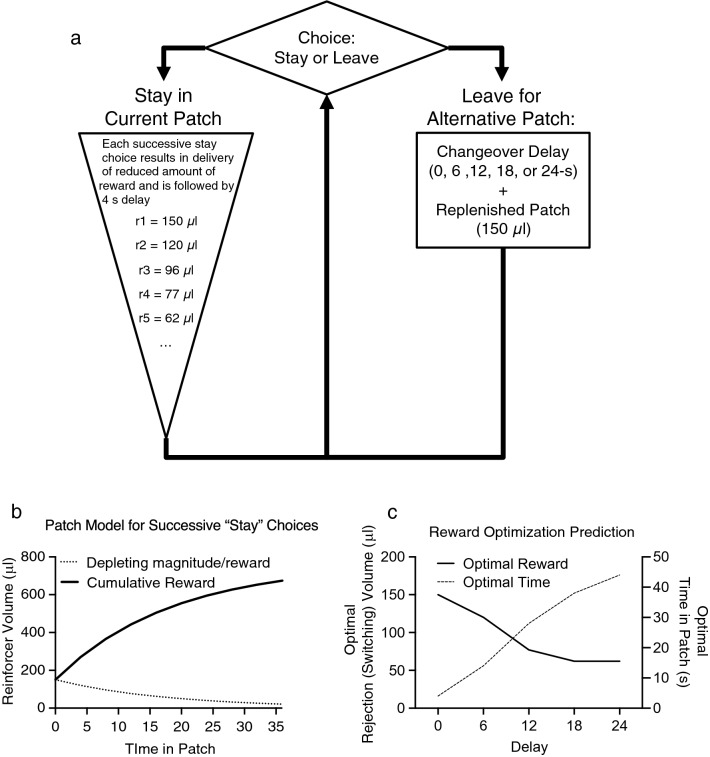
Schematic illustration of the sequential patch depletion procedure. (**a**) Rats are offered a sequential choice between two “patches” (snout poke receptacles). “Stay” choices (snout poke response to the same receptacle) resulted in presentation of decreasing volumes of water (r = reward; μl = microliters of water). “Leave” choices (snout poke response to the alternate receptacle) were followed by a changeover delay (travel cost) and presentation of the initial larger volume of water (150 μL). Following a “leave” choice, the abandoned patch is replenished to the original reward volume (150 μL). See text for full explanation. (**b**) Plot representing the volume of water across time in patch. The solid line represents the cumulative volume of water earned for successive stay choices in a patch. The dashed line represents the diminishing volume of water available for staying in the patch. (**c**) Plot representing the optimal rejection volume of water and time in patch across all delays tested according to the Marginal Value Theorem. The solid line represents optimal switching volume, whereas the dashed line represented optimal time spent in patch.

Behavior was measured using a sequential patch depletion procedure that was previously described (Fig. 6a)^26,83^. Briefly, during this task, water-restricted rats were offered a concurrent choice between two water “patches” (left- or back-wall snout poke receptacles) and could elect to “stay” in the current patch or “leave” for an alternative patch at any time.

A snout poke in the receptacle (i.e., entering the patch) resulted in immediate delivery of 150 µl water. If rats made a choice to “stay” in this patch, successively smaller amounts of water were delivered. To simulate patch depletion, the volume of water for successive “stay” choices was reduced 20% for each reinforcer presentation. For example, the first reward volume was 150 µl of water, the second reward was 120 µl, and the third reward was 96 µl, etc. with each reward delivery separated by a minimum of 4 s (Fig. 6a,b). Once in a patch, water was available according to a modified Fixed Interval (FI) 4-s schedule; each water delivery was followed by a 4-s interval during which the reinforcer was unavailable. However, successive “stay” choices were achieved in one of two ways: rats could emit a snout poke to the same receptacle as the previous response following the 4-s interval (traditional FI contingency) or the rat could remain with its snout in the receptacle for the duration of the 4-s interval (analogous to a Fixed Time or FT schedule).

If a rat made the choice to “leave” a patch by poking its snout in the alternative receptacle (“alternative patch”), a changeover delay (COD) was imposed to simulate “travel” cost^84^. During the COD, reinforcers were not available at either location regardless of responding. When the rat left a patch to travel to the alternative patch, the abandoned patch was replenished (the volume of the water was reset to 150 µl for the first reinforced response on returning to that patch).

When a patch change occurred with a 0-s COD, the stimulus light above the abandoned patch was extinguished, and the stimulus light above the newly poked receptacle was illuminated simultaneously with the delivery of 150 µl of water. For CODs > 0 s (6, 12, 18, or 24 s), the stimulus light above the abandoned receptacle was extinguished and a 1.9-kHz tone was pulsed for the duration of the COD. At the end of the delay, the pulsed tone was turned off and the stimulus light above the newly poked receptacle was illuminated. The first snout poke into the new location after the onset of the stimulus light resulted in the delivery of 150 µl of water. Sessions lasted for 10 min or until the rat earned a cumulative total of 5 ml of water, whichever occurred first.

The COD was constant within a session but varied between sessions (i.e., days of the week) in the following sequence (Monday through Saturday): 0, 0, 6, 12, 18, and 24 s, with the first session of the week being excluded from data analysis (i.e., the first 0 s session). This 6-day cycle was repeated four times for a total of 24 test sessions. Data from the last two cycles of sessions for each COD delay were averaged for data analysis.

### Dependent measures

There were five primary dependent measures: number of patch changes, average time in a patch, average water volume rejected by leaving the patch (“rejection volume”/“indifference point”), water consumption (μl/min/kg), and the deviation from optimality. The first and final patches, including associated patch changes, of each session were not included in these calculations. These patches were excluded because, in the case of the first patch, rats had not yet experienced the COD, and the last patch resulted in session termination due to the session ending and so not accurately representing rats’ choice behavior. Rate of reinforcement was controlled for by body weight and calculated by summing the volume of water earned and dividing that by time required to earn those reinforcers divided by body weight (kg). Number of patch changes was the number of times rats chose to switch to the alternative receptacle (“leave” choices) averaged over the last two sessions. Time in patch was the mean duration the rat stayed at one receptacle before leaving for the other. The rejection volume/indifference point was defined as the mean amount of water (µL) available at the abandoned patch when the rat switched to the alternative snout poke location, e.g. if the rat had earned 120 µl before leaving, the leaving volume would be the next volume scheduled for delivery, 96 µl in this example. The optimal rejection volume, based on the MVT, was operationally defined as the volume of water that maximized the average reward volume rate across all patches including the COD travel time. This was calculated as the cumulative rate of return from the patch (μL/s) for each reward, considering the COD length (travel time to the patch) and the 4 s between successive rewards (Fig. 6c). For example, when the COD was 6 s, the cumulative rate of return for the first reward in a patch was 150 μL divided by 6 s (25 μL/s). For the second reward, it was [150 + 120] μL divided by [6 + 4] s (27 μL/s). For the third reward, it was [150 + 120 + 96] μL divided by [6 + 4 + 4] s (26.14 μL/s). In this instance, an optimal strategy predicts leaving after two rewards are obtained and the rejection volume is 96 μL. The percent deviation from the optimal rejection volume (referred to as percent volume deviation) was calculated as follows: (optimal rejection volume − observed rejection volume)/optimal rejection volume × 100. Positive values indicate rats overharvested and stayed in the patch when the volume of the collected reinforcers had dropped below the value required to maximize, whereas negative values indicate the rat left when the volume was greater than the optimal volume. The same approach was used to calculate the percent deviation from optimal time in patch (referred to as percent time deviation) was calculated as follows: (observed time in patch − optimal time in patch)/optimal time in patch × 100. Positive values indicate the observed time in the patch was longer than optimal, and negative values indicate the observed time in the patch was less than optimal.

### Statistical analysis

Statistical analyses used SPSS Statistics software (IBM, Armonk, NY). Descriptive statistics indicated that the distributions of dependent measures were normal (skewness <|1|), so parametric statistics were used throughout.

To examine the effects of COD travel time on patch leaving, we used a mixed factor analysis of variance (ANOVA), with delay as the within-subject factor (0, 6, 12, 18, and 24 s) and sex as the between-subject factor (male and female), in conjunction with post-hoc comparisons using pairwise comparison Bonferroni test for within-subject comparisons and independent samples t-tests with Bonferroni corrections for between-subject comparisons. ANOVA effect sizes were reported as partial eta squared (**η**_**p**_^**2**^), and Cohen’s d (*d*) for post-hoc comparisons.

To examine whether analyses applied to traditional DD tasks was possible, hyperbolic equations were fitted to each rat’s rejection volume (indifference points), based on that described by Mazur^85^, using GraphPad Prism (GraphPad Software Inc., San Diego, CA):

$${\text{V}} = {\text{bA}}/\left( {{1} + {\text{kD}}} \right),$$

where V indicates the rejected volume of the diminishing reinforcer when the rat left the current patch for the alternative patch in µl, A represents the amount of water from the alternative patch (150 µL), and D represents the delay to receiving the 150-µL reinforcer (COD of 0, 2, 4, 8, 16, or 24 s). The bias parameter, b, was calculated such that the product of b and A equaled each animal’s indifference point at a 0-s delay^86^. The discount parameter (*k*) is an index for the rate of discounting or overall sensitivity to delayed reinforcers, such as the first reinforcer in an alternative patch. In DD tasks, larger values of k indicate steeper discount functions, stronger aversion to delayed reinforcers, more rapid devaluation of reinforcer value by delay, and thus greater impulsive choice. Here, k indicates higher relative levels of overharvesting and a preference for the smaller, sooner rewards available in the current patch over traveling to an alternative patch. The normalized area under the curve (AUC) of the discount function was calculated, which summarizes the influence of delay length on the choice to remain at a patch location. The AUC measure provides a simple measure of overharvesting/discounting that is not tied to a particular discount function^87^. Smaller AUC values indicate higher levels of overharvesting. The *k*, *b*, and AUC values were analyzed using independent samples t-tests, with sex as the between-subject factor.

Composite scores for all variables were calculated by the sum of the variable across all delays. These composite scores were used to calculate Pearson’s correlation coefficients. To assess the difference in the strength of their association with DD and reward maximization, correlation coefficients were compared using Fisher r-to-z transformations^32,33^, which is recommended for comparing correlation coefficients from the same sample with one variable in common^88^. For all statistical tests, a p < 0.05 was used as the alpha criterion.

## Supplementary Information

Supplementary Figure 1.

## Data Availability

The datasets used and/or analyzed during the current study available from the corresponding author on reasonable request.

## References

[1] Stephens DW, Brown JS, Ydenberg RC (2008). Foraging: Behavior and Ecology.

[2] Stephens DW, Krebs JR (2019). Foraging Theory.

[3] Charnov EL (1976). Optimal foraging, the marginal value theorem. Theor. Popul. Biol..

[4] Kendall RK, Wikenheiser AM (2022). Quitting while you’re ahead: Patch foraging and temporal cognition. Behav. Neurosci..

[5] Benson KE, Stephens DW (1996). Interruptions, tradeoffs, and temporal discounting. Am. Zool..

[6] McNamara JM, Houston AI (1987). A general framework for understanding the effects of variability and interruptions on foraging behaviour. Acta Biotheor..

[7] Carter EC, Redish AD (2016). Rats value time differently on equivalent foraging and delay-discounting tasks. J. Exp. Psychol. Gen..

[8] Constantino SM, Daw ND (2015). Learning the opportunity cost of time in a patch-foraging task. Cogn. Affect. Behav. Neurosci..

[9] Hayden BY, Pearson JM, Platt ML (2011). Neuronal basis of sequential foraging decisions in a patchy environment. Nat. Neurosci..

[10] Kane GA (2017). Increased locus coeruleus tonic activity causes disengagement from a patch-foraging task. Cogn. Affect. Behav. Neurosci..

[11] Kolling N, Behrens TE, Mars RB, Rushworth MF (2012). Neural mechanisms of foraging. Science.

[12] Nonacs P (2001). State dependent behavior and the marginal value theorem. Behav. Ecol..

[13] Shenhav A, Straccia MA, Cohen JD, Botvinick MM (2014). Anterior cingulate engagement in a foraging context reflects choice difficulty, not foraging value. Nat. Neurosci..

[14] Wikenheiser AM, Stephens DW, Redish AD (2013). Subjective costs drive overly patient foraging strategies in rats on an intertemporal foraging task. Proc. Natl. Acad. Sci..

[15] Sanchez-Roige S (2018). Genome-wide association study of delay discounting in 23,217 adult research participants of European ancestry. Nat. Neurosci..

[16] Coffey SF, Gudleski GD, Saladin ME, Brady KT (2003). Impulsivity and rapid discounting of delayed hypothetical rewards in cocaine-dependent individuals. Exp. Clin. Psychopharmacol..

[17] de Wit H (2009). Impulsivity as a determinant and consequence of drug use: A review of underlying processes. Addict. Biol..

[18] Jentsch JD (2014). Dissecting impulsivity and its relationships to drug addictions. Ann. N. Y. Acad. Sci..

[19] Johnson MW, Bruner NR, Johnson PS (2015). Cocaine dependent individuals discount future rewards more than future losses for both cocaine and monetary outcomes. Addict. Behav..

[20] Kirby KN, Petry NM (2004). Heroin and cocaine abusers have higher discount rates for delayed rewards than alcoholics or non-drug-using controls. Addiction.

[21] MacKillop J (2011). Delayed reward discounting and addictive behavior: A meta-analysis. Psychopharmacology.

[22] Perry JL, Carroll ME (2008). The role of impulsive behavior in drug abuse. Psychopharmacology.

[23] Reynolds B (2006). A review of delay-discounting research with humans: Relations to drug use and gambling. Behav. Pharmacol..

[24] Amlung M (2019). Delay discounting as a transdiagnostic process in psychiatric disorders: A meta-analysis. JAMA Psychiat..

[25] Bickel WK, Jarmolowicz DP, Mueller ET, Koffarnus MN, Gatchalian KM (2012). Excessive discounting of delayed reinforcers as a trans-disease process contributing to addiction and other disease-related vulnerabilities: Emerging evidence. Pharmacol. Ther..

[26] Richards JB (2013). Strong genetic influences on measures of behavioral-regulation among inbred rat strains. Genes Brain Behav..

[27] Parker CC (2014). Rats are the smart choice: Rationale for a renewed focus on rats in behavioral genetics. Neuropharmacology.

[28] Solberg Woods LC, Palmer AA (2019). Using heterogeneous stocks for fine-mapping genetically complex traits. Rat Genom..

[29] Chandler CM (2022). Effects of adolescent alcohol exposure via oral gavage on adult alcohol drinking and co-use of alcohol and nicotine in Sprague Dawley rats. Drug Alcohol Depend..

[30] McNamara TA, Ito R (2021). Relationship between voluntary ethanol drinking and approach-avoidance biases in the face of motivational conflict: Novel sex-dependent associations in rats. Psychopharmacology.

[31] Sherrill LK, Koss WA, Foreman ES, Gulley JM (2011). The effects of pre-pubertal gonadectomy and binge-like ethanol exposure during adolescence on ethanol drinking in adult male and female rats. Behav. Brain Res..

[32] Meng X-L, Rosenthal R, Rubin DB (1992). Comparing correlated correlation coefficients. Psychol. Bull..

[33] Diedenhofen B, Musch J (2015). cocor: A comprehensive solution for the statistical comparison of correlations. PLoS One.

[34] Zou GY (2007). Toward using confidence intervals to compare correlations. Psychol. Methods.

[35] Blanchard TC, Hayden BY (2015). Monkeys are more patient in a foraging task than in a standard intertemporal choice task. PLoS One.

[36] Hernandez CM (2020). Testicular hormones mediate robust sex differences in impulsive choice in rats. eLife.

[37] Van Haaren F, Van Hest A, Van de Poll NE (1988). Self-control in male and female rats. J. Exp. Anal. Behav..

[38] Koot S, van den Bos R, Adriani W, Laviola G (2009). Gender differences in delay-discounting under mild food restriction. Behav. Brain Res..

[39] Bayless DW, Darling JS, Daniel JM (2013). Mechanisms by which neonatal testosterone exposure mediates sex differences in impulsivity in prepubertal rats. Horm. Behav..

[40] Panfil K, Bailey C, Davis I, Mains A, Kirkpatrick K (2020). A time-based intervention to treat impulsivity in male and female rats. Behav. Brain Res..

[41] Lukkes JL, Thompson BS, Freund N, Andersen SL (2016). The developmental inter-relationships between activity, novelty preferences, and delay discounting in male and female rats. Dev. Psychobiol..

[42] Doremus-Fitzwater TL, Barreto M, Spear LP (2012). Age-related differences in impulsivity among adolescent and adult Sprague-Dawley rats. Behav. Neurosci..

[43] Eubig PA, Noe TE, Floresco SB, Sable JJ, Schantz SL (2014). Sex differences in response to amphetamine in adult Long-Evans rats performing a delay-discounting task. Pharmacol. Biochem. Behav..

[44] Perry JL, Stairs DJ, Bardo MT (2008). Impulsive choice and environmental enrichment: Effects of d-amphetamine and methylphenidate. Behav. Brain Res..

[45] Sackett DA, Moschak TM, Carelli RM (2019). Prelimbic cortical neurons track preferred reward value and reflect impulsive choice during delay discounting behavior. J. Neurosci..

[46] Becker JB, Hu M (2008). Sex differences in drug abuse. Front. Neuroendocrinol..

[47] Smith CL, Hantula DA (2008). Methodological considerations in the study of delay discounting in intertemporal choice: A comparison of tasks and modes. Behav. Res. Methods.

[48] Dittrich M, Leipold K (2014). Gender differences in time preferences. Econ. Lett..

[49] Kirby KN, Marakovic NN (1996). Delay-discounting probabilistic rewards: Rates decrease as amounts increase. Psychon. Bull. Rev..

[50] Stanovich KE, West RF, Toplak ME (2016). The Rationality Quotient: Toward a Test of Rational Thinking.

[51] Bembenutty H (2009). Academic delay of gratification, self-efficacy, and time management among academically unprepared college students. Psychol. Rep..

[52] Cross CP, Copping LT, Campbell A (2011). Sex differences in impulsivity: A meta-analysis. Psychol. Bull..

[53] Doidge JL, Flora DB, Toplak ME (2021). A meta-analytic review of sex differences on delay of gratification and temporal discounting tasks in ADHD and typically developing samples. J. Atten. Disord..

[54] Harrison EL, Coppola S, McKee SA (2009). Nicotine deprivation and trait impulsivity affect smokers' performance on cognitive tasks of inhibition and attention. Exp. Clin. Psychopharmacol..

[55] Logue AW, Anderson YD (2001). Higher-education administrators: When the future does not make a difference. Psychol. Sci..

[56] Prencipe A (2011). Development of hot and cool executive function during the transition to adolescence. J. Exp. Child Psychol..

[57] Reynolds B, Richards JB, Horn K, Karraker K (2004). Delay discounting and probability discounting as related to cigarette smoking status in adults. Behav. Process..

[58] Silverman IW (2003). Gender differences in delay of gratification: A meta-analysis. Sex Roles.

[59] van den Bos R, Homberg J, de Visser L (2013). A critical review of sex differences in decision-making tasks: Focus on the Iowa Gambling Task. Behav. Brain Res..

[60] Orsini CA, Setlow B (2017). Sex differences in animal models of decision making. J. Neurosci. Res..

[61] Westbrook SR, Hankosky ER, Dwyer MR, Gulley JM (2018). Age and sex differences in behavioral flexibility, sensitivity to reward value, and risky decision-making. Behav. Neurosci..

[62] Guajardo HM, Snyder K, Ho A, Valentino RJ (2017). Sex differences in μ-opioid receptor regulation of the rat locus coeruleus and their cognitive consequences. Neuropsychopharmacology.

[63] Ulloa R-E, Nicolini H, Fernández-Guasti A (2004). Sex differences on spontaneous alternation in prepubertal rats: Implications for an animal model of obsessive-compulsive disorder. Prog. Neuropsychopharmacol. Biol. Psychiatry.

[64] Baran SE, Armstrong CE, Niren DC, Conrad CD (2010). Prefrontal cortex lesions and sex differences in fear extinction and perseveration. Learn. Mem..

[65] Grafe LA, Cornfeld A, Luz S, Valentino R, Bhatnagar S (2017). Orexins mediate sex differences in the stress response and in cognitive flexibility. Biol. Psychiat..

[66] Ridley RM (1994). The psychology of perserverative and stereotyped behaviour. Prog. Neurobiol..

[67] Hoyenga KB, Hoyenga KT (1982). Gender and energy balance: Sex differences in adaptations for feast and famine. Physiol. Behav..

[68] Key C, Ross C (1999). Sex differences in energy expenditure in non–human primates. Proc. R. Soc. Lond. Ser. B.

[69] Blanchard TC, Pearson JM, Hayden BY (2013). Postreward delays and systematic biases in measures of animal temporal discounting. Proc. Natl. Acad. Sci. U. S. A..

[70] Tropp J, Markus EJ (2001). Sex differences in the dynamics of cue utilization and exploratory behavior. Behav. Brain Res..

[71] Pellman BA, Schuessler BP, Tellakat M, Kim JJ (2017). Sexually dimorphic risk mitigation strategies in rats. Eneuro.

[72] Islas-Preciado D (2020). Risk-based decision making in rats: modulation by sex and amphetamine. Horm. Behav..

[73] Orsini CA, Willis ML, Gilbert RJ, Bizon JL, Setlow B (2016). Sex differences in a rat model of risky decision making. Behav. Neurosci..

[74] Yates JR (2021). Differential effects of glutamate N-methyl-d-aspartate receptor antagonists on risky choice as assessed in the risky decision task. Psychopharmacology.

[75] Carroll ME, Kohl EA, Johnson KM, LaNasa RM (2013). Increased impulsive choice for saccharin during PCP withdrawal in female monkeys: Influence of menstrual cycle phase. Psychopharmacology.

[76] Liley AE, Gabriel DBK, Sable HJ, Simon NW (2019). Sex differences and effects of predictive cues on delayed punishment discounting. eNeuro.

[77] Mazur JE, Vaughan W (1987). Molar optimization versus delayed reinforcement as explanations of choice between fixed-ratio and progressive-ratio schedules. J. Exp. Anal. Behav..

[78] Hackenberg TD, Axtell SA (1993). Humans'choices in situations of time-based diminishing returns. J. Exp. Anal. Behav..

[79] Sodetz, F. J. Appetitive and Aversive Schedule Preferences: Schedule Transitions as lntervening Events. *The Effect of Delay and of Intervening Events on Reinforcement Value: Quantitative Analyses of Behavior, Volume V*, 141 (2013).

[80] Hansen C, Spuhler K (1984). Development of the National Institutes of Health Genetically Heterogeneous Rat Stock. Alcoholism.

[81] Du Sert NP (2020). Reporting animal research: Explanation and elaboration for the ARRIVE guidelines 2.0. PLoS Boil..

[82] Lloyd DR, Kausch MA, Gancarz AM, Beyley LJ, Richards JB (2012). Effects of novelty and methamphetamine on conditioned and sensory reinforcement. Behav. Brain Res..

[83] Ishiwari, K. et al. *Delay Discounting Measured Using a Sequential Patch Depletion Procedure*. https://www.protocols.io/view/delay-discounting-measured-using-a-sequential-patc-n92ldzqnnv5b/v1

[84] Herrnstein RJ (1970). On the law of effect. J. Exp. Anal. Behav..

[85] Mazur JE (1987). The Effect of Delay and of Intervening Events on Reinforcement Value. Quantitative Analyses of Behavior.

[86] Richards JB, Mitchell SH, De Wit H, Seiden LS (1997). Determination of discount functions in rats with an adjusting-amount procedure. J. Exp. Anal. Behav..

[87] Myerson J, Green L, Warusawitharana M (2001). Area under the curve as a measure of discounting. J. Exp. Anal. Behav..

[88] Weaver B, Wuensch KL (2013). SPSS and SAS programs for comparing Pearson correlations and OLS regression coefficients. Behav. Res. Methods.

