# Supplementary material for: Reward maximization assessed using a sequential patch depletion task in a large sample of heterogeneous stock rats
Source: Sci Rep. 2023 Apr 29;13:7027. doi: 10.1038/s41598-023-34179-8 (PMC10148848; doi:10.1038/s41598-023-34179-8)
Supplement: Supplementary file 1 — Supplementary Figure 1. [file 41598_2023_34179_MOESM1_ESM.docx]

**Supplementary Figure 1.** Area Under the Curve (AUC) for Patch Utilization variables (a) This plot represents the normalized AUC of water consumption (μl/min/kg) as a function of delay. Females exhibited significantly higher AUCs compared to males. (b) This plot represents the normalized AUC of number of patch changes as a function of delay, with males exhibiting significantly higher AUCs compared to females. (c) This plot represents the normalized AUC of time in patch as a function of delay. Females exhibited significantly higher AUCs compared to males.

* p < 0.05, ** p < 0.01, *** p < 0.001 between males (n = 896) and females (n = 898).
